# Prediction of lameness using automatically recorded activity, behavior and production data in post-parturient Irish dairy cows

**DOI:** 10.1186/s13620-021-00182-6

**Published:** 2021-02-06

**Authors:** G. M. Borghart, L. E. O’Grady, J. R. Somers

**Affiliations:** 1 University College Dublin, Dublin, Ireland; 2 Glanbia Ireland, Kilkenny, Ireland

**Keywords:** Lameness, Dairy cow, Supervised classification, Machine learning, Accelerometer

## Abstract

**Background:**

Although visual locomotion scoring is inexpensive and simplistic, it is also time consuming and subjective. Automated lameness detection methods have been developed to replace the visual locomotion scoring and aid in early and accurate detection. Several types of sensors are measuring traits such as activity, lying behavior or temperature. Previous studies on automatic lameness detection have been unable to achieve high accuracy in combination with practical implementation in a on farm commercial setting. The objective of our research was to develop a prediction model for lameness in dairy cattle using a combination of remote sensor technology and other animal records that will translate sensor data into easy to interpret classified locomotion information for the farmer. During an 11-month period, data from 164 Holstein-Friesian dairy cows were gathered, housed at an Irish research farm. A neck-mounted accelerometer was used to gather behavioral metrics, additional automatically recorded data consisted of milk production and live weight. Locomotion scoring data were manually recorded, using a one-to-five scale (1 = non-lame, 5 = severely lame). Locomotion scores where then used to label the cows as sound (locomotion score 1) or unsound (locomotion score ≥ 2). Four supervised classification models, using a gradient boosted decision tree machine learning algorithm, were constructed to investigate whether cows could be classified as sound or unsound. Data available for model building included behavioral metrics, milk production and animal characteristics.

**Results:**

The resulting models were constructed using various combinations of the data sources. The accuracy of the models was then compared using confusion matrices, receiver-operator characteristic curves and calibration plots. The model which achieved the highest performance according to the accuracy measures, was the model combining all the available data, resulting in an area under the curve of 85% and a sensitivity and specificity of 78%.

**Conclusion:**

These results show that 85% of this model’s predictions were correct in identifying cows as sound or unsound, showing that the use of a neck-mounted accelerometer, in combination with production and other animal data, has potential to replace visual locomotion scoring as lameness detection method in dairy cows.

## Introduction

With the ongoing growth of the world population and the abolition of the EU milk quotas in 2015, opportunities for the expansion of the dairy sector persist. Efficiency, and thereby the milk production per cow, has been increased over the past decades by selective breeding, increased milking frequency and feeding [18]. This intensification, together with specialization, has had implications on animal welfare. Restricted opportunity to perform natural behaviors such as grazing and increased pressure on staff time has led to a higher prevalence of both lameness and mastitis [17, 19, 37]. Earlier studies have reported lameness prevalence varying from 1.2% [53] to 5.1% in Sweden [41] to 36% in England and Wales [10] and between 23 and 70% in Europe and North America [30].

Lameness is an expression of pain, which can have several causes including trauma, infectious diseases and disfunction of one or more hooves or limbs [13, 52, 67]. This diverse range of disorders and their multifactorial etiology, make lameness difficult to prevent and treat, resulting in potentially a major welfare issue on farms [13, 48]. Bovines instinctively mask pain, lowering visual expression of lameness and impeding visual detection [20, 24, 45]. Early detection of lameness can increase treatment success, may prevent lameness from becoming chronic and may diminish the negative impact of lameness on production [8, 21, 71].

Lameness is usually detected by the herdsman, claw trimmer or veterinarian, during routine trimming or by visual inspection of the cow’s locomotion. Visual locomotion scoring (LS) is time consuming and subjective, and farmers currently have less time to intensely monitor herd health due to increased farm size [6, 50]. Consequently, lameness detection and treatment are often delayed, with Alawneh et al. [2] estimating a median interval of 28 days from the onset of a LS > 3, recorded using the 5-point scale described by Sprecher et al. [55], to the treatment of lameness.

Because visual observation of the gait of cows has limits, other indicators of lameness may be useful for detection. Examples of these indicators include a change in the behavioral time-budget of cows, weight shifting between hind legs and resting of a painful foot [26, 58]. Sepúlveda-Varas et al. [51] found a relation between increase in beta-hydroxybutyrate (BHBA) concentrations and development of lesions in cows. Several studies have attempted to use these indicators to predict the degree and onset of lameness [36, 50, 72].

Automated detection methods for lameness have been developed to aid in early and accurate detection of lameness. Several types of sensors are involved in these methods, to measure traits as activity, lying behavior and temperature [7, 8]. However, scarcely any of the researched methods and models have been implemented in practice, mostly due to excessive error rates and false positives alerts, or costs and ease of implementation for the technology [32, 60]. Zhao et al. [71] investigated if gait characteristics could be extracted from leg swing analyses, using computer vision, and this resulted to be effective for quantifying lameness degeneration. However, it was suggested to explore a combination of technologies to generate a combined system that outputs a continuous locomotion score. Another study researched the possibility of downscaling the Gaitwise pressure mat, without significantly losing detection performance of lameness [60]. The study concluded that when both the measurement-zone length and sensor resolution were reduced, the same performance was achieved compared to the original set up. Wood et al. [70] reported that an increase in temperature associated with foot lesions could be detected by using thermography, but the lesions could not be differentiated with the technique. Thorup et al. [57] compared symmetries of left and right limb pair curves using force plates and demonstrated lower levels of symmetry to be present in lame cows compared to non-lame cows.

A study by Haladjian et al. [28] used a support vector machine classification algorithm to classify cow’s strides as normal or abnormal. Anomaly detection was used, which aims to learn a computing device what “normal” events look like to detect deviations from those, in their case lameness. Another study used a decision tree for the classification of lameness into 3 categories, after data was gathered using an automatic vision-based system [71].. A decision tree uses if-then rules at each split of a branch to label all the items with a certain class in each leaf [71]. Jiang et al. [33] used a deep learning network for the detection of key parts of dairy cows, which can be used in lameness detection by using video analysis technology.

Given the challenge outlined above, there is a high demand for high performance, and easy to implement, analytical detection models that translate sensor data into useful information for farmers.

The objectives of this study were to use machine learning methods to develop a prediction model for lameness in dairy cattle, using commercially available remote sensor technology in combination with routinely available animal data translated into classified lameness predictions, useful for the farmer to replace visual locomotion scoring for early lameness detection.

## Materials and methods

The study was carried out in accordance with University College Dublin’s (UCD) guidelines and approval on ethical animal research.

From February 2017 until December 2017, data were collected from 164 Holstein-Friesian dairy cows, housed at the UCD Lyons Research Farm, Newcastle, Co. Dublin, Ireland. The cows were milked twice daily in a 45-unit rotary parlor, in which they were fed supplemental concentrates based on individual energy requirements. The farm operated a pasture-based production system with daytime grazing during February and October/November and full-time grazing from March until October. During the winter months, the cows were fed a grass silage-based diet. Locomotion scores (LS) were recorded manually, by a trained veterinarian (JS) according to Sprecher et al. [55], which is based on a one-to-five scale, with one being non-lame and five being severely lame. LS assessment of all the milking cows was performed weekly on a 30-m stretch of solid concrete floor, when the cows exited the parlor after milking in the afternoon.

All the cows at the farm received a routine foot trimming of the hind feet at the start of the study in February 2017. The Dutch 5-step method [59] was used to trim all cows’ hind feet, since most lesions affect the hind claws [11, 54], front feet were examined when indicated through lameness assessment. Footbaths containing an 8% copper sulphate solution were used routinely during the study period. The footbath routine consisted of two walk-through treatment footbaths, preceded by a single plain water footbath, that were placed along the exit corridor of the milking parlor. The cows walked through these baths after morning and evening milking, three non-consecutive days per week, for every three weeks.

A neck-mounted accelerometer (MooMonitor+®, Dairymaster®, Causeway, Ireland) continuously recorded activity of the cows, using a 3-dimensional accelerometer which determined the cow movement and head direction. The accelerometer data were classified into 6 behavioral metrics, consisting of 3 activity metrics (low, medium, high), rumination, resting and feeding, based on a commercial algorithm designed for grazing dairy cow systems [29, 65]. The thresholds for the 3 levels of activity were based on activity intensity. Every 15 min, the number of minutes spent on each behavior category was registered in the MooMonitor+. Additional data available for each cow were lactation number and milk production data: milk yield (kg), milk constituents (fat/protein/lactose) and somatic cell count. Live weight data were recorded weekly, throughout lactation, using an electronic weighing platform (Dairymaster®, Causeway, Ireland).

### Data analysis

Before the data was analyzed, data preparation was performed for several variables in the datasets. The behavioral metrics were recorded on 15 min basis and were averaged as minutes per hour. When there were no milk production and/or live weight records available on the day of LS, an average was calculated from the recording before and after the LS. To investigate patterns of change over time, lagged variables were created for the variables going back in time 21 days.

The final data set consisted of 3799 behavioral observations with associated live weight and milk production data. All analyses were conducted using R version 3.4.2 [47]. Basic descriptive statistics were calculated using the “psych” package [49]. Given the objective of identifying potentially lame animals, cows which received LS 1 were labelled as “sound”, cows with LS ≥ 2 were labelled as “unsound”. This wording, instead of “lame” and” non-lame”, was chosen to create a more balanced data set, since there were not so many cows with LS > 3.

Machine learning methods were used to develop a range of predictive models. Supervised classification analysis was used to investigate whether cows could be classified as sound and unsound, based on all available behavioral activity, production and animal data, using the assigned label based on LS as reference. Four classification models were built, each containing a different combination of the available data, which were trained on a random subset of 60% of the data set and tested on the remaining 40% of the data set. The training set is bigger to prevent overfitting. The first model contained the 6 MooMonitor+ behavioral metrics, the second model contained lactation and DIM data. The third model consisted of the data from the first and second model combined, and the fourth model consisted of the same data as the third but also added live weight and milk production data (Table 1).

**Table 1:** Overview of the data included in the four classification models that were built

Model	Data included
1	MooMonitor+ behavioral metrics
2	Lactation number and DIM
3	Lactation number, DIM and MooMonitor+ behavioral metrics
4	Lactation number, DIM, MooMonitor+ behavioral metrics, live weight and milk production data^a^

^a^Milk production data consisted of milk yield (kg), milk constituents (fat/protein/lactose) and somatic cell count

A gradient boosted decision tree machine learning algorithm, xgboost [16] was used for classification of sound and unsound cows. To improve the accuracy of each predictive model, hyperparameter tuning was performed by doing a grid search, which was set up manually, and 10-fold cross-validation was used as resampling method. The packages “caret” [68] and “xgboost” [16] were used in R to build these models.

These four models were constructed with a default cut-off value > 0.5 predicted probability for classification of a cow as unsound. An optimal cut-off value for each model was determined to maximize predictive specificity and sensitivity using Youden’s index [23].

Using a confusion matrix, the prediction output of the classification was evaluated against the assigned class based on LS as reference [40]. The true-positive (TP / (TP + FN)) and true-negative (TN / (TN + FP)) rates can be obtained from the confusion matrix, as well as the Cohen’s kappa statistic. The kappa statistic measures how well the classifier has performed as compared to how well it would have performed by chance. Models achieving kappa statistic values between 0 and 0.20 are interpreted as “slight”, between 0.21 and 0.40 represent “fair” models, between 0.41 and 0.60 represent “moderate” models, between 0.61 and 0.80 represent “substantial” models, between 0.81 and 0.99 represent “almost perfect” models, and a value of 1 represent a model which shows perfect agreement between predicted and observed classification [5, 39, 69]. A receiver-operator characteristic curve (ROC-curve) was plotted to check the diagnostic ability of the classification model at different threshold values [23]. Accuracy was evaluated by the area under the curve (AUC). As second method to measure accuracy, a calibration plot was built [22, 62]. A well-calibrated model has a calibration curve that “hugs” the straight line (y = x), corresponding to true probability (LS) equals to predicted probability of unsound cows in our study. At last, variable importance was evaluated per model using variable importance plots. Importance is ascribed to every variable in the model by measuring the improvement of its role as substitute to the primary split in the model, using AUC as measure [9, 38]. The importance’s of the variables are then scaled to have a maximum of hundred.

## Results

The number of recordings with LS 1 were 1979 (52.1%), with LS 2 were 1086 (28.6%), with LS 3 were 544 (14.3%), with LS 4 were 185 (4.9%) and with LS 5 were 5 (0.1%) over the whole study period. This resulted in 1820 (47.9%) unsound labelled recordings in the complete data set. The differences in the behavioral metrics, milk yield and live weight between sound and unsound cows, displayed in Table 2, were small and the standard deviations were large.

**Table 2:** Mean, median and standard deviations per MooMonitor+ behavioral metric, milk yield and live weight for sound and unsound labelled cows in the merged data set (3799 recordings; 1979 sound and 1820 unsound)

Variable (min/hour/day)	Group	N	Mean	Median	Sd
Rumination	Sound	1979	20.55	20.66	4.06
	Unsound	1820	20.44	20.55	4.21
Resting	Sound	1979	15.81	15.36	4.94
	Unsound	1820	16.99	16.31	5.46
Feeding	Sound	1979	19.27	20.72	6.26
	Unsound	1820	18.18	19.26	6.44
Activity^a^					
Low	Sound	1979	2.46	1.64	2.22
	Unsound	1820	2.32	1.78	1.72
Medium	Sound	1979	1.53	1.27	1.35
	Unsound	1820	1.62	1.31	1.52
High	Sound	1979	0.38	0.21	0.86
	Unsound	1820	0.46	0.20	1.08
Milk yield	Sound	1979	24.39	23.85	8.29
	Unsound	1820	27.81	28.00	9.84
Live weight	Sound	1979	620.61	618.5	76.1
	Unsound	1820	683.45	681.5	76.68

^a^The activity data from the accelerometer consisted of 3 levels (low, medium, high), based on a commercial algorithm designed for grazing dairy cow systems

The kappa statistic of the fourth model was the closest to 1 (Table 3), which represents a model that shows moderate agreement between predictions and observations [39, 69]. The first model has the lowest kappa value (closer to zero, Table 3). A kappa value close to zero, means that the model did not perform better than chance [69].

**Table 3:** Overview of the kappa (with 95% CI), accuracy, specificity, sensitivity and area under the curve (AUC) per model, with the default cut-off value (0.5)

Model	Kappa [95% CI]	Accuracy	Specificity	Sensitivity	AUC
1	0.14 [0.09–0.19]	57%	60%	53%	0.61
2	0.53 [0.48–0.57]	75%	74%	77%	0.81
3	0.55 [0.51–0.59]	76%	76%	76%	0.84
4	0.58 [0.54–0.62]	78%	78%	78%	0.85

The first model consisted of MooMonitor + behavioral metrics. The second model contained data on lactation and DIM, the third model added the behavioral metrics to that, and the fourth model added live weight and production data on top of the data of the third model

The highest performance was achieved with the fourth model (AUC = 0.85), which contained the most variables in the model, using the default cut-off value (Table 3). A model with only the 6 behavioral metrics was the least performing model (AUC = 0.61). The trend that was observed in the performance of the models, was that with increasing number of variables the performance increased (Table 3).

**Fig. 1:**
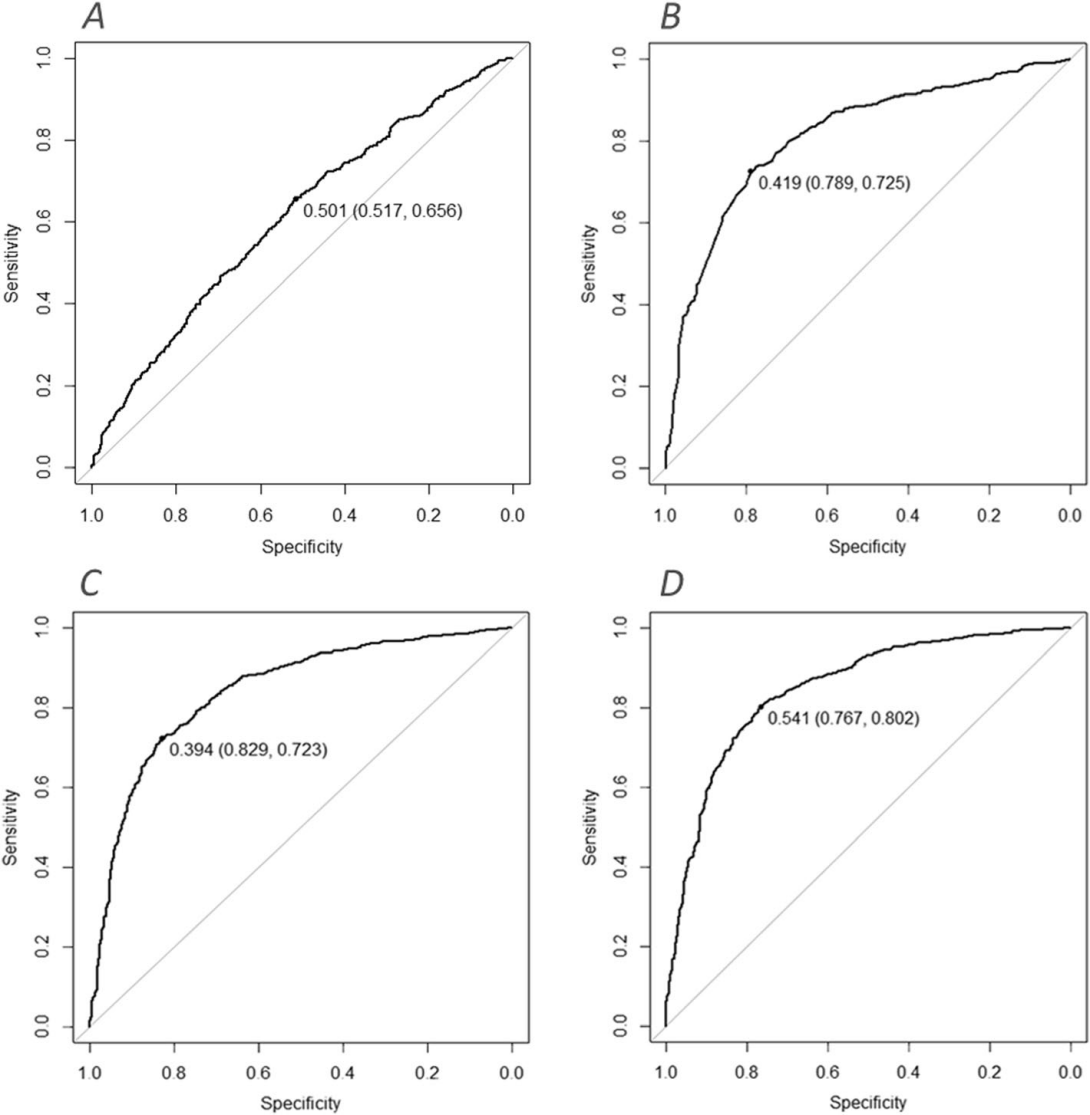
ROC-curves with the optimal cut-off value and associated specificity and sensitivity between brackets, for the first model **a**, containing the 6 MooMonitor+ behavioral metrics, the second model **b**, containing lactation number and DIM data, for the third model **c**, consisting of the 6 MooMonitor+ behavioral metrics and lactation and DIM and for the fourth model **d**, consisting of the 6 MooMonitor+ behavioral metrics, lactation, DIM, live weight and milk production data

With the ROC-curve, the optimal cut-off value was determined for each model, using Youden’s index. For the first model, the optimal cut-off value resulted to be 0.5, the associated specificity 0.52 and the sensitivity 0.66 (Fig. 1a). The second model achieved an optimal cut-off of 0.42, with a specificity of 0.79 and sensitivity of 0.73 (Fig. 1b). The optimal cut-off of the third model was 0.39, the specificity 0.83 and the associated sensitivity was 0.72 (Fig. 1c). For the fourth model the optimal cut-off was 0.54, the associated specificity 0.77 and sensitivity 0.80 (Fig. 1d).

**Fig. 2:**
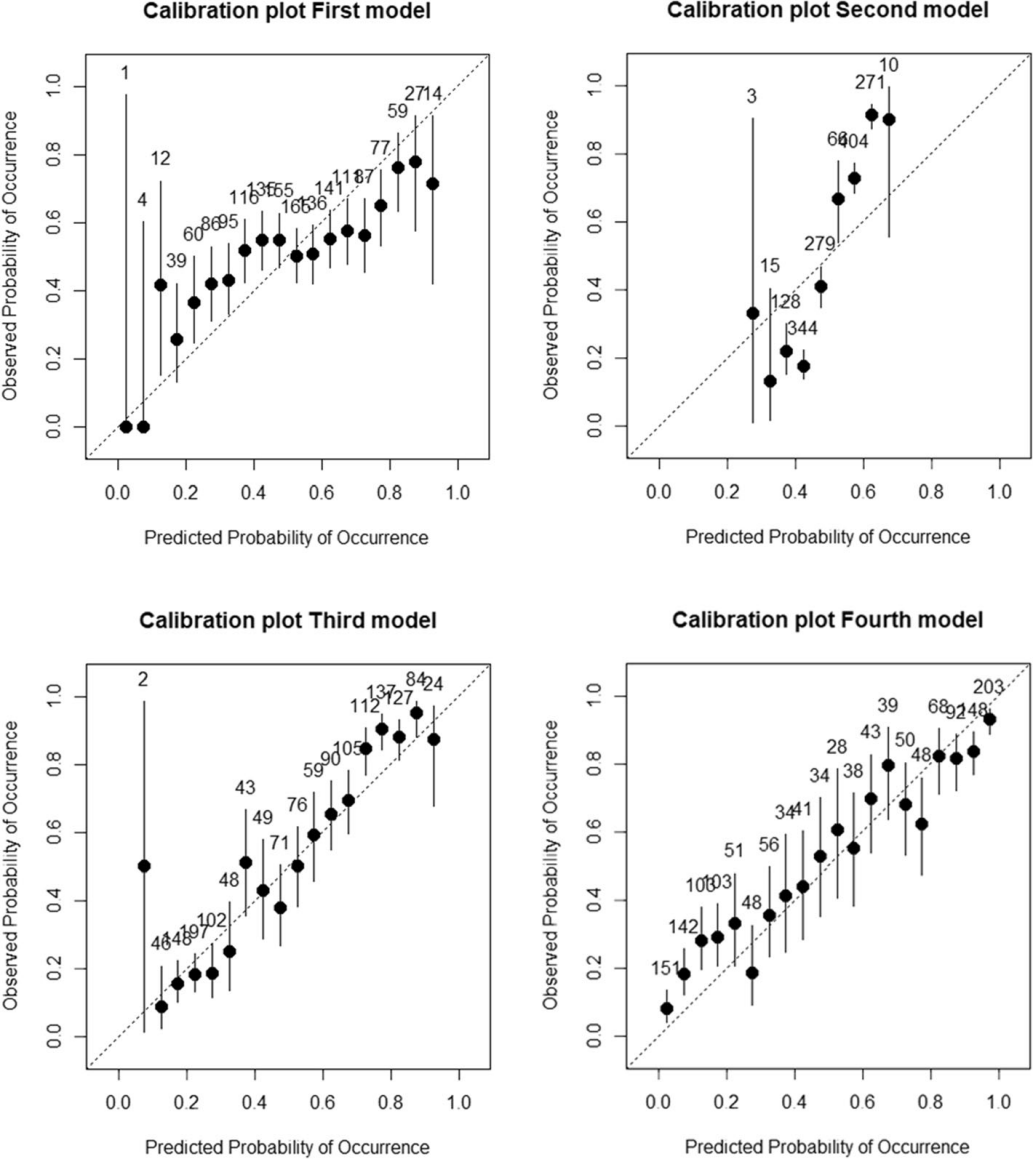
Calibration plots for the first model, containing the MooMonitor+ behavioral metrics, the second model, containing lactation number and DIM, the third model, containing lactation number, DIM and MooMonitor+ behavioral metrics, and the fourth model, containing lactation number, DIM, MooMonitor+ behavioral metrics, live weight and production data. The number above the points in the plot indicate the number of records each prediction group contains, based on the predicted probabilities of the recordings. The lines show the 95% CI. A well-calibrated model has the points close to the diagonal line, indicating agreement between predicted and observed probabilities

The accuracy of the four models was additionally checked using calibration plots (Fig. 2). The calibration plots for the third and fourth model show good calibration, since most of the points were close to the straight line (Fig. 2).

Changing from the first to the second model, keeping the cut-off value equal, increased the accuracy from 57 to 75%. Also, the specificity (+ 14%), sensitivity (+ 24%), and the AUC (from 0.61 to 0.81) increased. The differences between the second and third model were smaller (around 1% increase/decrease), which was similar for the difference between the third and fourth model. However, there was a bigger impact of changing from the second to the third model visible in the calibration plot, meaning an increase in reliability of the predictions of the third model.

**Fig. 3:**
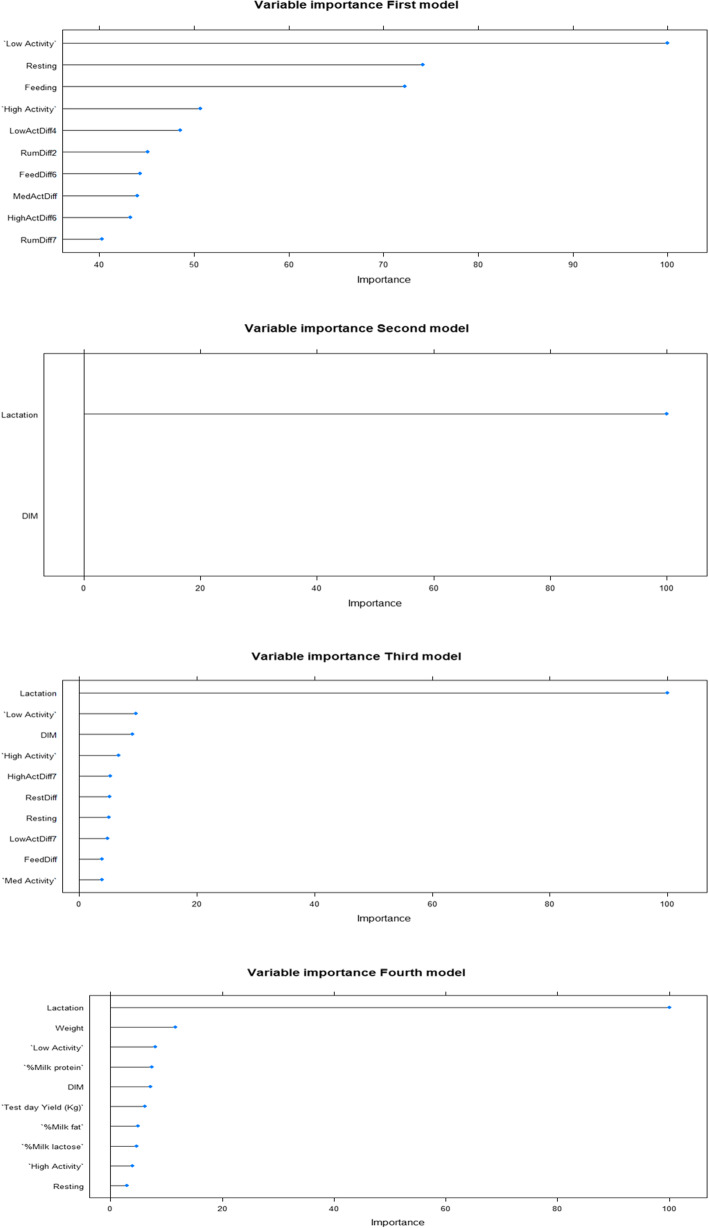
Variable importance plots for the first model, containing the MooMonitor+ behavioral metrics, the second model, containing lactation number and DIM, the third model, containing lactation number, DIM and MooMonitor+ behavioral metrics, and the fourth model, containing lactation number, DIM, MooMonitor+ behavioral metrics, live weight and production data. In the plots, variables with “Diff” and a number are the lagged variables going back in time the amount of days as the number in the name states. Importance is ascribed to each variable by using AUC. In the plots, the importances are scaled to have a maximum of hundred

At last, the importance of each variable to the models was visualized in variable importance plots. For the first model, which contained the MooMonitor+ behavioral metrics, the most important variables were the low activity level, resting and feeding. For the second, third and fourth models, the variable lactation was the most important, followed by DIM, liveweight and the low activity level (Fig. 3).

## Discussion

Based on information from sensor data, provided by a neck mounted accelerometer, LS were used to classify cows into sound or unsound. This was a suitable solution to the small number of cows with extreme LS values. Additionally, we considered it more important that all potentially lame cows were identified for examination by the farmer rather than trying to determine the precise score of each cow. If lameness is detected and treated in an early stage, the development of a chronic lameness state may be avoided [27, 46, 66].

Previous studies have used different methods for the analysis of sensor data. A study performed to develop a method for automatic classification of accelerometer data into dairy cow behaviors used support vector machines, but this method has a considerable computational cost [42]. Another study used decision tree induction to detect clinical mastitis with sensor data and found that the model improved by using boosting [35]. A regression tree with a boosting technique based on additive logistic regression was used for lameness detection, but detection performance was not high enough for practical implementation [34]. Therefore, the type of supervised classification model used in this study, extreme gradient boosting, was chosen to achieve high levels of both speed and performance [44].

The small difference in accuracy between the third and fourth model may be explained by the fact that the impact of body weight on lameness remains unclear. Some studies suggested that body weight is possibly resulting from lameness and is not a causative factor [63, 64]. However, other studies found a significant effect of live weight loss on lameness occurrence [3, 4]. The first model consisted only of activity data, which performed less powerful in classifying cows as sound and unsound. This study showed that inclusion of activity data alone into classification models for lameness detection achieved lower accuracy compared to models that included a combination of activity data, production data, lactation number and DIM as data inputs, or a model that only included lactation and DIM. These results support the findings of Chapinal et al. [15], Blackie et al. [12] and Ito et al. [31], which reported that a combination of several variables, such as weight distribution, lying bout duration and walking speed, was most promising for automated lameness detection.

Classification models with AUC values around 0.85, which the third and fourth models achieve, can be interpreted as good models (0.80 > AUC < 0.90) according to Swets [56]. These models achieved higher AUC values compared to prediction models in other studies using the same combination of data, such as the studies performed by Chapinal et al. [15], Kamphuis et al. [34], Van Hertem et al. [61], which achieved AUC values up to 0.75, and Miekley et al. [43] up to 0.8. The model with the highest sensitivity was the fourth model. This sensitivity was higher compared to other studies which used a comparable combination of data [25, 34, 61].

The optimal cut-off values, with their associated specificities and sensitivities, were obtained with ROC-curves by determining the cut-off values where the sum of sensitivity and specificity was maximal [1]. With the optimal cut-off value, the fourth model obtained the highest sensitivity, which means that this model was the best in not missing out possible lame cows. Higher sensitivities are of great interest when the models are used to detect treatable diseases. The study by Haladjian et al. [28], which used an algorithm for the detection of anomalies in walking patterns of cows, achieved a sensitivity of 74.2%. Our approach thus achieved a better result for detecting possible lame cows, while they achieved a higher specificity. The third model achieved the highest specificity with the optimal cut-off value, which means that this model has the probability of causing the farmer to examine of too many non-lame cows. The specificity of this model was higher than the specificity achieved in a study that used radar data, which were analyzed with a machine learning algorithm to automatically classify the cows as lame or non-lame [14].

## Conclusions

The present study analyzed the possibility to use machine learning methods to develop a prediction model for lameness in dairy cattle, using commercially available remote sensor technology in combination with routinely available animal data. Our results showed that the use of the MooMonitor+ for the detection of unsound cows was best when used in combination with lactation number, DIM, milk production and live weight data, resulting in a high degree of predictive accuracy. The application of this technology has the potential to be highly beneficial in reducing the time to lameness diagnosis and treatment and therefore making a significant impact on animal welfare. Further research should involve testing the external validity of the models in commercial pasture based dairy production systems. In addition, the association of lameness causing lesions and the predicted classification should be explored.

## Data Availability

The datasets generated and/or analysed during the current study are not publicly available due to data sharing restrictions. For further enquiries please contact the corresponding author.

## Abbreviations

AUC: Area under the curve
BHBA: Beta-hydroxybutyrate
CI: Confidence interval
DIM: Days in milk
EU: European Union
FN: False negative
FP: False positive
LS: Locomotion score
ROC: Receiver operator cuve
TN: True negative
TP: True positive
UCD: University College Dublin
